# Supplementary material for: Real-time observation of a correlation-driven sub 3 fs charge migration in ionised adenine
Source: Commun Chem. 2021 May 20;4:73. doi: 10.1038/s42004-021-00510-5 (PMC9814501; doi:10.1038/s42004-021-00510-5)
Supplement: Supplementary file 1 — Supplementary Information [file 42004_2021_510_MOESM1_ESM.pdf]

Supplementary Information

This file contains: Supplementary Methods (sections 1–11), Supplementary Figures S1–S18, Supplementary Table S1 and Supplementary References 34–53.

Supplementary Methods

1. Experimental set-up and data acquisition

Carrier-envelope-phase stable laser pulses with 4 fs duration, 2.5 mJ energy and a central wavelength of approximately 700 nm are used to drive the attosecond pump–probe setup[10,34,35]. The beamline is based on a Mach–Zehnder-type interferometer where one arm (70 % of the initial energy) is used for XUV generation while the other arm provides the NIR probe pulse at adjustable delays. Collinear recombination of the two beams is achieved with a drilled mirror reflecting the IR and transmitting the XUV in the central hole. Sub-300 as XUV pulses are generated in krypton or xenon (depending on the desired cut-off energy) by polarisation gating[19]. A typical spectrum generated in krypton is shown in Figure S1. A 100-nm-thick aluminium filter is used to remove the residual NIR (as well as harmonics below 15 eV) from the XUV arm. The attosecond interferometer is actively delay-stabilised with a residual RMS of 20 as.

Adenine powder (Sigma-Aldrich, >99 %) is evaporated at 463 K in a resistively heated stainless steel oven with a flow of helium acting as carrier and buffer cooling gas. According to vibrational spectroscopy on adenine from a similar source, the lowest-energy tautomer (9H amino) dominates[36]. The jet passes through a 1-mm diameter skimmer and down to the optical interaction region of a standard velocity map imaging spectrometer[37] operated as a time of flight mass spectrometer. The voltages of the microchannel plate (MCP) and phosphor screen are gated to avoid detecting the helium gas at short times of flight.

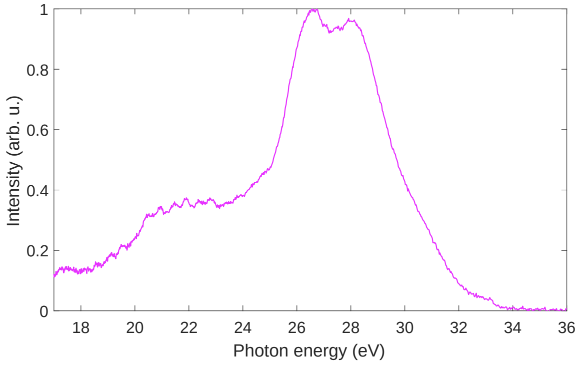

Figure S1. XUV spectrum of isolated attosecond pulse generated in krypton.

Pump–probe data were acquired by scanning the delay in alternating directions over multiple traces (7 in the main dataset used, 5 for the atomic time reference scan). Each trace was normalised to the total yield to account for a slow sample depletion. These multiple traces have been used to calculate the errorbars shown for the experimental signal, as a shaded grey area in Fig. 1b, Figure S6 and Figure S7.

2. Fragmentation probability

To estimate the fraction of XUV-ionized adenine molecules that dissociate we can compute the fraction of ions with lower mass than adenine, within the mass range detectable with the gated MCP operation. Background gases could however exaggerate this fraction, for instance by nitrogen gas having the same molecular mass as the H_2CN^+ fragment (at 28 u/e in Figure S2). We therefore separate the XUV-only mass spectrum into sample and background components using the following generic method:

Two mass spectra, $y_{\text{full}}(m)$ and $y_{\text{reduced}}(m)$, need to be recorded with identical conditions (e.g. XUV spectrum and residual gas density) but with different density of the adenine sample, achieved by changing the oven temperature. We use a scaling factor $0 < x < 1$ to represent the remaining adenine density in y_{reduced} with respect to the full density in y_{full}:

$$\begin{cases} y_{\text{full}}(m) &= y_{\text{background}}(m) + y_{\text{sample}}(m) \\ y_{\text{reduced}}(m) &= y_{\text{background}}(m) + x\, y_{\text{sample}}(m) \end{cases} \quad (1)$$

To determine the scaling factor, a region of the mass spectrum is defined as background-free, e.g. the region covering the parent cation. Solving equation (1) for x in the background-free region, where $y_{\text{background}}(m) = 0, m \in M_{\text{parent}}$, gives

$$x = \sum_{m\in M_{\text{parent}}} y_{\text{reduced}}(m) \Bigg/ \sum_{m\in M_{\text{parent}}} y_{\text{full}}(m) \quad (2)$$

$$y_{\text{background}}(m) = \frac{y_{\text{reduced}}(m) - x y_{\text{full}}(m)}{1-x} \quad (3)$$

$$y_{\text{sample}}(m) = y_{\text{full}}(m) - y_{\text{background}}(m) \quad (4)$$

As the separation is based on the difference between two mass spectra, the output contains some noise and artefacts. A simple correction that was made was to require both the background and sample signals to be non-negative. After evaluating (3), $y_{\text{background}}$ is

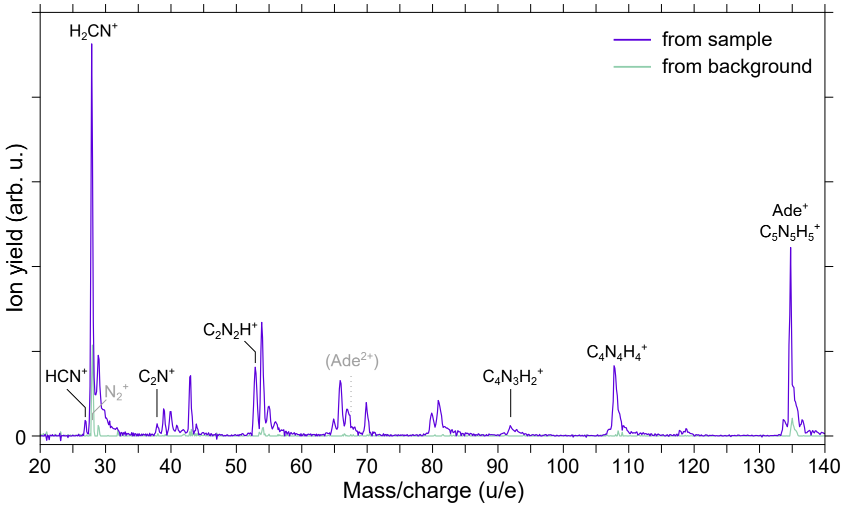

Figure S2. **Separation of background and adenine contributions to the XUV mass spectrum.** Of the raw peak at 28 u/e, 16% was deduced to be N_2^+ from the background instead of $HCNH^+$ from adenine. The dotted line indicates where Ade^{2+} would appear (67.5 u/e), but it is essentially absent without NIR probe (compare Figure S3).

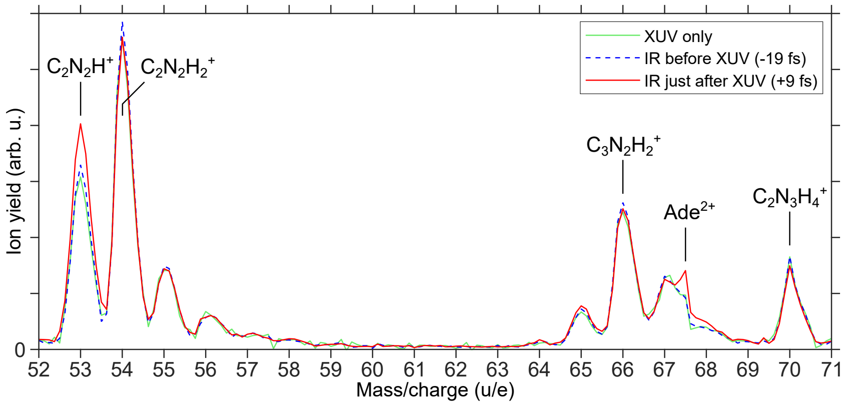

Figure S3. **Appearance of the adenine dication.** Zoom-in on the mass spectrum where the dication clearly appears at a small positive NIR delay but is negligible by the XUV alone or with the NIR at a negative delay.

updated at individual mass/charge-bins m to ensure that $0 \leq y_{\mathrm{background}}(m) \leq y_{\mathrm{full}}(m)$. The latter inequality ensures that also the final y_{sample} remains non-negative when computed as in (4).

The resulting separated adenine and background mass spectra are shown in Figure S2 for masses above 20 u/e. In this range, the adenine mass spectrum consists of 81% fragments and 19% parent cation. The background gas removal procedure was not applied to pump–probe data, as it was introduced for the sole purpose of estimating the total fragment yield.

3. Adenine dication formation as a function of the XUV photon energy

We show that the stable adenine dication yield is maximised when the NIR probe pulse is sent at a small but nonzero delay after the XUV pump. As a complement to the delay-dependence curves of individual ions in Fig. 1b we show mass spectra at negative and positive NIR delay in Figure S3, together with the XUV-only case. Since sending the NIR pulse before the XUV pulse gives the same result as the XUV only case, we conclude that there is no NIR-pump–XUV-probe contribution.

At 67.5 u/e, where the dication appears, there is also a background contribution due to the width of the adjacent fragment at 67 u/e. Since the unambiguous part of the 67 u/e peak shows no delay-dependence, this additional constant background will not have any influence on the results concerning delay-dependence (such as Fig. 1b). Estimating the absolute yield of the dication, however, requires the background to be subtracted. From the perturbed curve shape, we es-

timated a 0.2 % production of adenine dication with XUV only, which we consider almost negligible.

In Figure S4 we examine the effect of limiting the XUV photon energy on the pump–probe dynamics. The three panels show the NIR-induced change in the yields of the two cationic fragments with the largest absolute change as well as the adenine dication. Solid lines correspond to the normal case where the XUV photon energy of the attosecond pulses extends from 15 to 35 eV, employing XUV generation in Kr and an aluminium filter. Dotted lines show a scan where the XUV generated in Xe is limited to photon energies below 17 eV by an indium filter (about 5.2 eV below the dication ground state). In this last case, the absorption of one XUV photon and a few NIR photons is not able to produce any stable dication. Consequently, we could conclude that population of the special state (identified as responsible of the stabilisation process) can be only observed if ionisation occurs using the higher-energy part of the XUV spectrum. We also note that with the reduced cut-off, many fragments disappear, even the largest NIR-induced absolute step for the 53 u/e fragment. For 108 u/e there may still be a negative step, but close to the noise level.

4. Number of absorbed NIR photons

A sequence of short scans were made over a range of NIR intensities from approximately $7{\times}10^{12}$ to $1.4{\times}10^{13}$ W/cm^2 to estimate the number of NIR photons involved in the XUV+NIR process(es) producing each detected ion. We first extracted the step height, h_m, of the pump-probe signal for each ion (at mass/charge) at different NIR intensities. We then fitted the NIR-intensity-dependent data with the power law $h_m(I) = (c_m I)^{n_m}$, where I represents the NIR intensity and n_m represents the number of NIR photons driving the process in addition to the single XUV photon (see top panels of Figure S5). Although the interpretation of n_m is straightforward for the parent cation and dication, one must for a fragment ion see it as a net result of the NIR probe's competing enhancement (via dissociation of the parent and larger fragments) and depletion (via further dissociation) of the signal. We acknowledge that there is some uncertainty (possibly 25 %) in the absolute NIR intensities in Figure S5, but a linear rescaling of the intensities would not affect the determination of the nonlinearity order n_m, only the coefficient c_m.

The results of the fitting procedure are reported in the bottom panel of Figure S5: the step heights of the small fragments scale approximately linearly while the adenine dication's step height scales approximately quadratically with the NIR intensity. This result suggests that 2 NIR photons are required in the probing step (after shake-up) of the process we discuss in the main manuscript, yielding a non-dissociated doubly charged adenine dication.

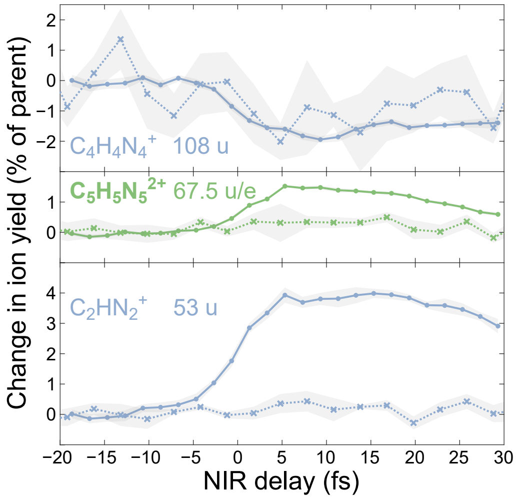

Figure S4. Pump-probe results for different XUV photon energies. Pump–probe scan signals are shown in solid lines for the main scan (35 eV cut-off) and dotted lines for indium-filtered HHG from xenon (17 eV cut-off).

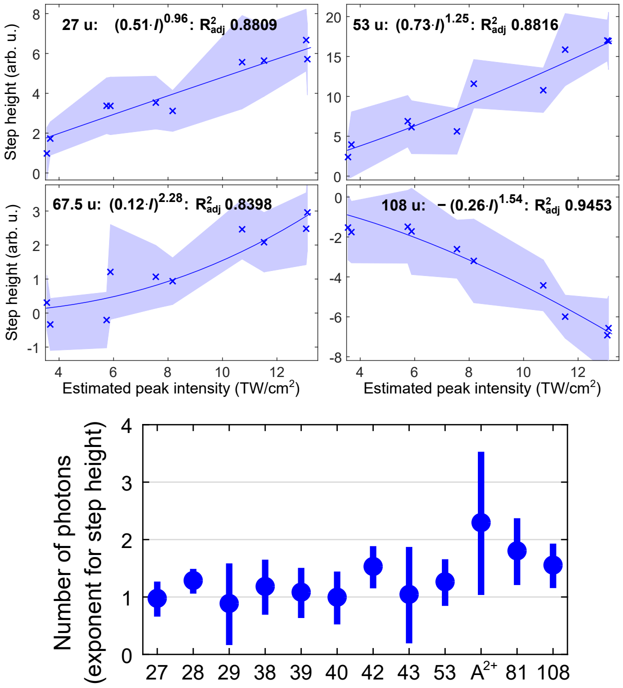

Figure S5. Scaling with NIR intensity. The delay-dependent step height scales linearly with NIR intensity for several small fragments but approximatively quadratically for the adenine dication. Vertical bars and areas show the fitter's estimated 68 % confidence intervals.

5. Fitting of pump–probe signals

When an initial state populates the probed state at rate $1/\tau_1$, and the probed state decays with the rate $1/\tau_2$, the general solution to the rate equations allows the population of the probed state to be expressed as:

$$N(t) = \begin{cases} N_1 \frac{1}{1-\tau_1/\tau_2}\left(\mathrm{e}^{-t/\tau_2} - \mathrm{e}^{-t/\tau_1}\right) & , t > 0 \\ 0 & , t < 0 \end{cases} \quad (5)$$

under the condition that $0 \leq \tau_1 < \tau_2$, i.e. the state population will rise to a positive peak and then decay. Here, t is the pump–probe delay axis of the scan, positive when the NIR pulse comes after the XUV attosecond pulse. An arbitrary coefficient N_1 is included for generality.

To model experimental pump–probe scans, the population function should be convoluted with an instrument response function and the temporal overlap of the XUV and NIR pulses needs to be defined with a t_0-parameter. We allow for a constant background level b and name the step height parameter h. Since convolution is a linear operation, we can convolve the two terms in (5) separately and express the complete curve model as

$$f(t) = b + \frac{h}{1-\tau_1/\tau_2}\left(g_{\tau_2}(t-t_0) - g_{\tau_1}(t-t_0)\right). \quad (6)$$

Here, $g_\tau(t)$ represents the convolution of a single exponentially decaying step function with a Gaussian instrument response function[24] of full-width at half-maximum $W = 2\sqrt{2\ln 2}\sigma_{\mathrm{IRF}}$. The convolution is evaluated using the expression[38]

$$g_\tau(t) = \frac{1}{2}\mathrm{erfc}\left(\frac{\sigma_{\mathrm{IRF}}}{\tau\sqrt{2}} - \frac{t}{\sigma_{\mathrm{IRF}}\sqrt{2}}\right) \times \exp\left(-t/\tau + \sigma_{\mathrm{IRF}}^2/(2\tau^2)\right) \quad (7)$$

and normalized ($\int_{-\infty}^{\infty} g_\tau(t)\mathrm{d}t = \tau$) such that the step height before convolution is 1.

For an initial overview, we employed the simplest possible scheme where separate fits were made for each ion, with independent values for t_0 and W, and with simple step functions ($\tau_1 = 0$). From the result in Figure S6(a), it is clear that a later t_0-parameter is needed for the dication. We compute $\Delta t_0 = t_0(\mathrm{Ad}^{2+}) - \bar{t_0}(\text{reference cations}) = 1.82 \pm 0.33$ fs. A classical time-difference like Δt_0 is however not physically meaningful. To obtain a result which can be compared with the theoretically extracted shake-up time, we introduced a risetime of the adenine dication signal, by using a variable τ_1 in the function for Ade^{2+}. The t_0-parameter is now common for all ions and defines the zero of the NIR-delay axis, and for robustness we use a common W-parameter and perform a global fit of all the shown ions. The dication and the 108 u/e fragment where assigned as 2-photon processes for the best fit (in agreement with section 4), meaning that their Gaussian width was $W/\sqrt{2}$ instead of W since a *two*-photon signal scales with the *square* of NIR intensity envelope. The results in Figure S6(b)

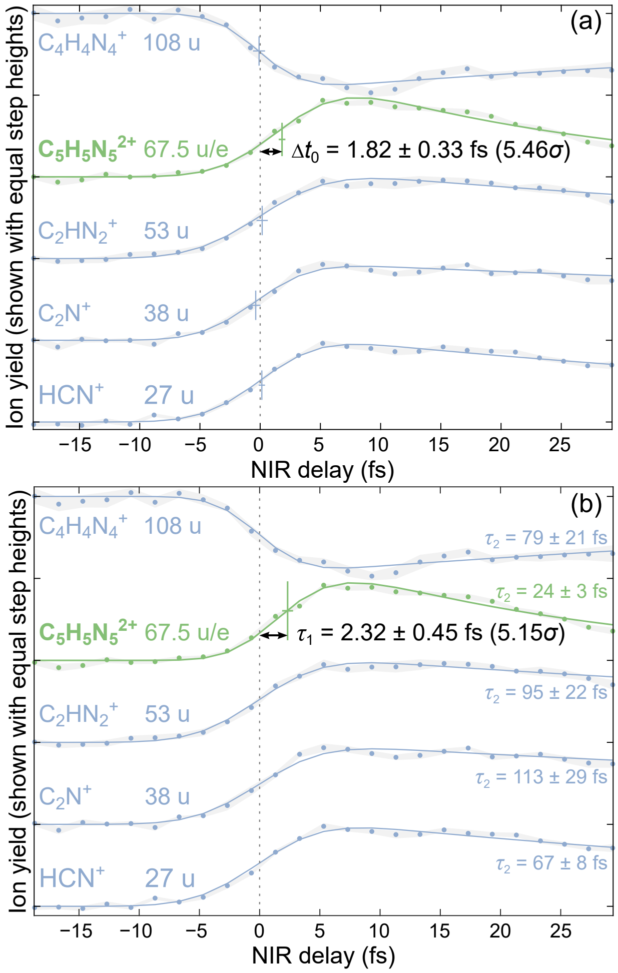

Figure S6. **Pump–probe scan fitted using different approaches**. (a) Simple step functions (having $\tau_1 = 0$) at individually variable t_0 were fitted without constraints. We find that the adenine dication ($C_5N_5H_5^{2+}$, 67.5 u/e) has a distinct delay with respect to the weighted t_0-average of the cationic fragments, and show the NIR-delay axis relative to the cation average. (b) To obtain the more theoretically meaningful risetime of the adenine dication signal, τ_1 was included in the function for the dication. The t_0-parameter to define the zero of the NIR-delay axis is now common for all ions, and for robustness we use a common W-parameter fitted to 9.9 ± 0.5 fs. The dication and the 108 u/e fragment where assigned as 2-photon processes for the best fit (in agreement with section 4), meaning that their Gaussian width was $W/\sqrt{2}$ instead of W. Panel (b) corresponds to Fig. 1b in the main article.

show a significantly nonzero risetime for the dication $\tau_1 = 2.32 \pm 0.45$ fs, which we can now compare to the theoretically extracted shake-up time (see main text).

6. Zeroing of the delay axis

In our main analysis we discuss the delay of the adenine dication under the assumption that the steps of many cations occur directly at the temporal overlap of XUV and NIR pulses. Although this is is the natural assumption when so many fragments appear synchronised, one could still speculate that some common molecular motion is required before the NIR probe influences any cation fragment yield, shifting all steps to a positive NIR delay. To rule this possibility out, we performed another experiment where a small amount of the atomic gas krypton was simultaneously injected into the vacuum chamber with adenine. The Kr^{2+} yield in Figure S7 exhibits a sharp step (2-photon process meaning width parameter $W/\sqrt{2}$) for which any molecular dynamics can be excluded. For simplicity, we used only the first fitting procedure described in the previous section to extract a t_0-parameter for Kr^{2+} to be compared with the other cationic fragments. The results of the fitting are shown in Figure S7. We find that the Kr^{2+} step almost coincides with the steps for the adenine fragments and that Ade^{2+} is the only significantly delayed ion.

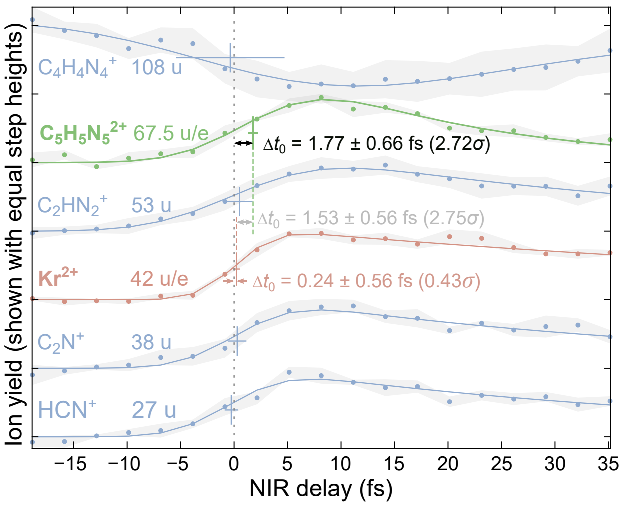

Figure S7. Pump–probe scan with Kr^{2+} (42 u/e) as atomic time reference. Three Δt_0 parameters, extracted using the first fitting procedure described in the previous section, are displayed: the difference between Ade^{2+} and the reference cations (black text), the difference between Ade^{2+} and Kr^{2+} (grey text) and the difference between Kr^{2+} and the reference cations (red text). Ade^{2+} is significantly delayed while the cationic fragments are simultaneous with the Kr^{2+} reference.

7. Calculating equilibrium properties

Ground state ab-initio characterisation of the Adenine molecule (sketched in Fig. S8) shows that its double ionized species has a stable geometry as long as it is allowed to relax. The effect of the geometry relaxation on the charged states of Adenine is relatively small as it can be seen from Fig. S9 for the singly (a) and doubly (b) ionised case. In such a figure, we report the relative variation (with respect to the optimised neutral structure) of the bond lengths at each optimisation step. We quantify the effect of geometry relaxation in terms of the total energy difference of singly (Ade^+) and doubly charged (Ade^{2+}) state with respect to the neutral (Ade) state. The values are reported in Tab. S1 for the unrelaxed and relaxed structures. We can infer that the minimum energy that the XUV+IR pulses have to transfer to the system in order to reach the dication with a stable geometry is ~ 15 eV, which is in agreement with the experimental observation that no dication is observed if the high energy part of the XUV spectrum is filtered out.

The calculations have been performed with the Octopus code[27] within a DFT framework using the PBE

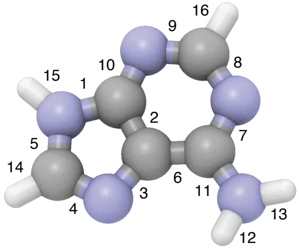

Figure S8. Adenine molecule bond numbering

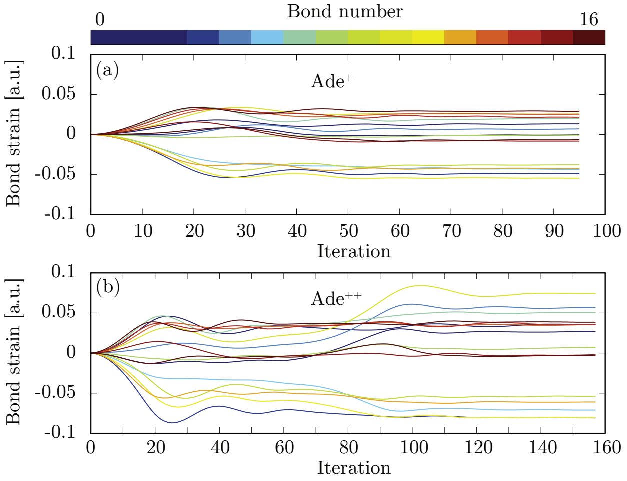

Figure S9. Geometry relaxation. relative variation of the bond length as a function of the optimisation steps for singly (a) and doubly (b) ionised adenine. The reference bond length is the one for the neutral state.

Table S1. Ground State Energy differences

$E^{1^+} - E^0$	$E^{2^+} - E^0$	$E^{1^+}_{\rm rel} - E^0$	$E^{2^+}_{\rm rel} - E^0$
8.17 eV	22.21 eV	1.88 eV	14.99 eV

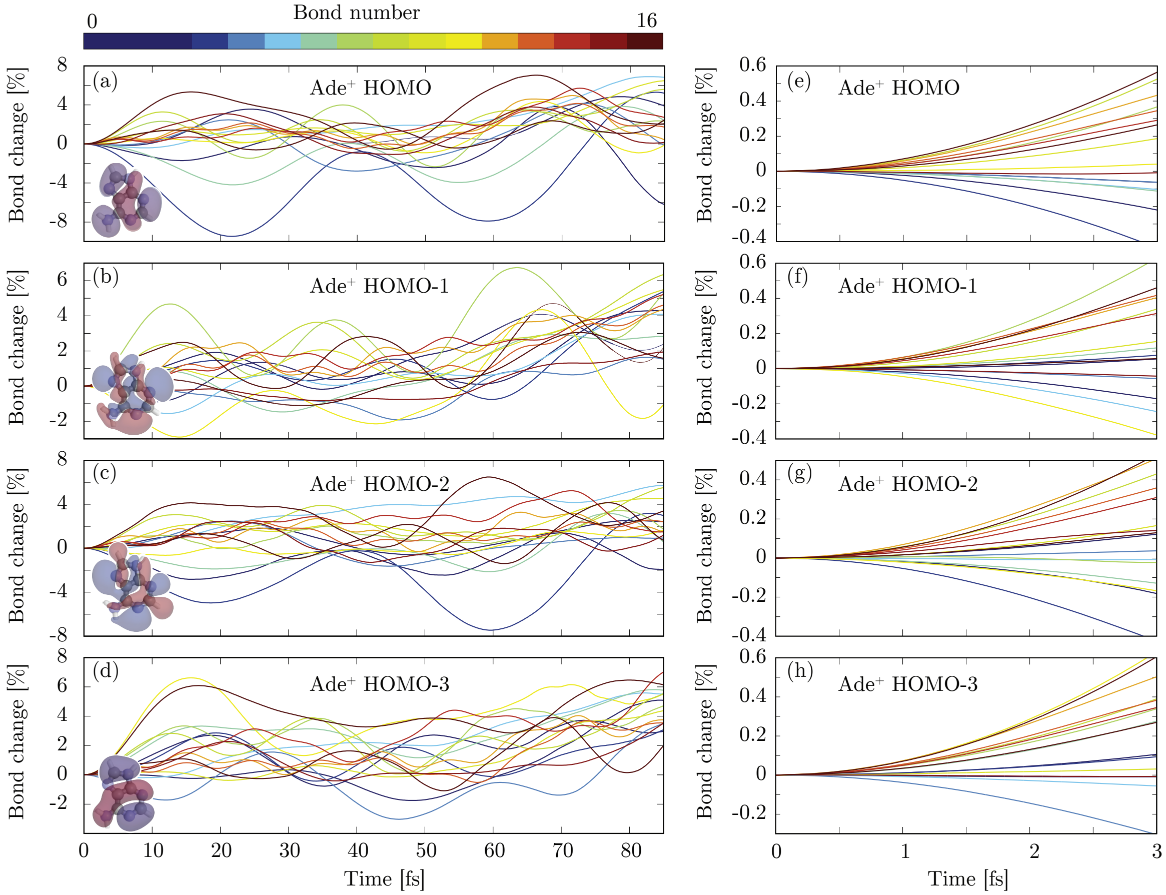

Figure S10. **Bond length variations following sudden ionisation with TDDFT**. Adenine cations are time propagated following a sudden removal of an electron from different orbitals of the cation Ade$^+$, (a,e) HOMO, (b,f) HOMO-1, (c,g) HOMO-2, (d,h) HOMO-3. In all situations it appears that sudden ionisation leads to substantial bond elongation and potential fragmentation at long times. In all the cases, negligible bond elongation is observed in the first 3 fs. Illustrations of the wavefunction from which the electron is removed are shown on the left side of panels (a)–(d).

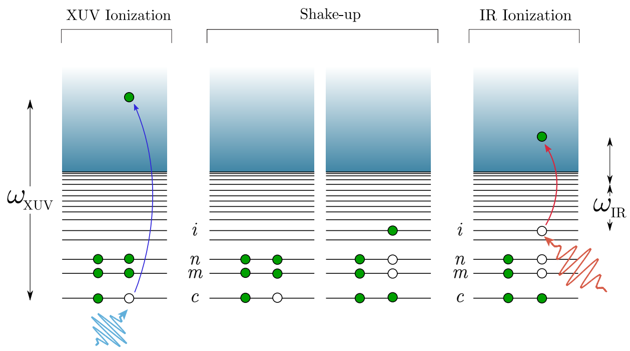

Figure S11. **Three step model for the creation of stable adenine dication**. The molecule is ionised by the XUV pulse leaving it with hole in the core state c. During the shake-up process, this core-hole moves up to the valence region, through the excitation of an electron from the occupied level n (or m) to the unoccupied level i and the refilling of the core hole by an electron from level m (or n). Finally the IR pulse ionises an electron from this excited level, leaving the molecule doubly ionised.

functional together with the so called averaged density self-interaction correction (ADSIC)[39] which corrects the Coulomb potential tails. A grid spacing of $h = 0.28$ Bohr has been used for the simulations.

8. Bond elongation

In this section we demonstrate that ionisation of adenine leads to substantial bond elongation and therefore possible dissociation on the long time-scale (> 10 fs) but no considerable effect is observed in the time scale of the charge migration process. For such a proof we perform a sudden single and double ionisation and we let the electronic and nuclear system propagate in time by means of TDDFT+Ehrenfest dynamics[30,40]. In our calculations, the removal of electrons is orbital specific, in Fig. S10(a)–(d) we show the bond length dynamics for singly ionised adenine where the electron has been suddenly removed at the beginning of the time propagation from a given orbital. In all the outer valence orbitals investigated, the ionisation leads to substantial bond elongation. We note, however, that in all cases, bond elongation is negligible in the first 3 fs after ionisation, as shown in the zoom-in panels.

9. Modelling the internal electronic relaxation process with rate equations

a. Shake-up matrix elements

Owing to electron-electron correlation an ionised system can undergo an electronic rearrangement, in which previously unoccupied bound states become populated through a so called shake-up process, which is depicted in the central panel of Fig. S11.

Driven by Coulomb interaction, the electron in ϕ_m fills the hole in ϕ_c and transfers the energy to the electron in ϕ_n which is then excited to the state ϕ_i. Overall, the shake-up process has to conserve energy, i.e. $\epsilon_i - \epsilon_n = \epsilon_m - \epsilon_c$. Here we are interested in calculating the rate for such a process for the adenine molecule following XUV photoionisation. Using Fermi's golden rule such a rate is given by

$$W_{FI} = 2\pi |\langle \Psi_F | \hat{V}_C | \Psi_I \rangle|^2 \delta(\epsilon_F - \epsilon_I), \tag{8}$$

where Ψ_I and Ψ_F are the initial and final many-body wavefunctions, $\hat{V}_C$ the Coulomb interaction operator and the Dirac delta ensures energy conservation. Let us first restrict our analysis to the four specific single particle states $\phi_c, \phi_m, \phi_n, \phi_i$. In such a basis, the Coulomb interaction, that we consider as the perturbation that drives the shake-up, can be written (in second quantization) as:

$$\hat{V}^C_{mn} = \frac{1}{2} \sum_{\sigma\sigma' \in \{\uparrow\downarrow\}} \Big\{ v_{cimn} c^\dagger_{c\sigma} c^\dagger_{i\sigma'} c_{m\sigma'} c_{n\sigma} + v_{icmn} c^\dagger_{i\sigma} c^\dagger_{c\sigma'} c_{m\sigma'} c_{n\sigma} + h.c. \Big\}, \tag{9}$$

where we have defined the Coulomb integrals as:

$$v_{ijmn} = \int d\mathbf{r} d\mathbf{r}' \phi^*_i(\mathbf{r}) \phi^*_j(\mathbf{r}') \frac{1}{|\mathbf{r} - \mathbf{r}'|} \phi_m(\mathbf{r}') \phi_n(\mathbf{r}). \tag{10}$$

In our picture, the initial state of a shake-up process is the one resulting from the XUV photo-ionisation and, without loss of generality, we choose it to be $|\Psi_I\rangle = c_{n\uparrow}|\Psi_0\rangle$, with $|\Psi_0\rangle$ consisting of doubly occupied i, m and n orbitals. The application of the Coulomb perturbation on such an initial state can be evaluated as follows:

$$\begin{aligned} \hat{V}^C_{mn} |\Psi_I\rangle &= \frac{1}{2} \sum_{\sigma\sigma' \in \{\uparrow\downarrow\}} \Big\{ v_{cimn} c^\dagger_{c\sigma} c^\dagger_{i\sigma'} c_{m\sigma'} c_{n\sigma} + v_{icmn} c^\dagger_{i\sigma} c^\dagger_{c\sigma'} c_{m\sigma'} c_{n\sigma} + h.c. \Big\} c_{n\uparrow} |\Psi_0\rangle \\ &= \frac{1}{2} \sum_{\sigma \in \{\uparrow\downarrow\}} \Big\{ v_{cimn} c^\dagger_{i\sigma} c^\dagger_{c\uparrow} c_{n\uparrow} c_{m\sigma} + v_{icmn} c^\dagger_{i\sigma} c^\dagger_{c\uparrow} c_{m\uparrow} c_{n\sigma} \Big\} c_{n\uparrow} |\Psi_0\rangle, \end{aligned} \tag{11}$$

where the h.c. does not contribute because it contains terms of the type $c^\dagger_{m\sigma}c^\dagger_{n\sigma'}$. Knowing the result of the Coulomb operator on the initial state and assuming the final state to be $|\Psi_F\rangle = |\Psi_i^{mn}\rangle = c^\dagger_{i\uparrow}c_{m\uparrow}c_{n\uparrow}|\Psi_0\rangle$ (again, without loss of generality) we can apply the Fermi golden rule in Eq. (8) and write:

$$R^{\mathrm{Sh-up}}_{imnc} = \frac{\pi}{2}\ |v_{icmn} + v_{cimn}|^2\,\delta(\epsilon_i - \epsilon_n - (\epsilon_m - \epsilon_c)). \tag{12}$$

In general, for a given initial core-hole state c and a final occupied excited state i there might be several m and n state combinations compatible with the shake-up process. For this reason the rate of the shake-up independent of the orbital localization of the two final holes is given by:

$$R^{\mathrm{Sh-up}}_{ic} = \frac{\pi}{2}\sum_{n\geq m}|v_{icmn} + v_{cimn}|^2\,\delta(\epsilon_i - \epsilon_n - (\epsilon_m - \epsilon_c)). \tag{13}$$

Note that we restricted the sum to $n \geq m$ to avoid the double counting that would arise from the fact that the holes are identical. For practical purposes the delta function in the formula above is replaced by a Lorentzian, $L^{\gamma_j}_{\epsilon_j}(\omega) = \frac{1}{2\pi}\frac{\gamma_j}{(\omega-\epsilon_j)^2+(\gamma_j/2)^2}$, as follows:

$$R^{\mathrm{Sh-up}}_{ic} = \frac{\pi}{2}\sum_{n\geq m}|v_{icmn} + v_{cimn}|^2\,L^{\eta}_{\epsilon_m+\epsilon_n-\epsilon_i-\epsilon_c}(\omega=0), \tag{14}$$

where the width η enters as a parameter. In the following we choose $\eta = 0.1$ eV and a discussion on how the results depend on such value is presented in subsection (c).

b. Ionisation rate: evaluating orbital dependent cross-sections

The initial state in the shake-up process is created by the XUV pulse (see leftmost panel of Fig. S11) and the probability of creating the initial hole in a state ϕ_i is determined by the XUV photoionisation probability of such a state which is given by:

$$P^{\mathrm{Ion}}_i(\omega) = 2\pi n_{\mathrm{spin}}\sum_{\mathbf{k}}\left|\langle\phi_{\mathbf{k}}|e^{i\mathbf{q}\cdot\hat{r}}|\phi_i\rangle\right|^2\delta(\omega - (\epsilon_{\mathbf{k}} - \epsilon_i))$$
$$= \frac{2\pi}{V}\sum_{\mathbf{k}}\left|\int d\mathbf{r}e^{i(\mathbf{k}+\mathbf{q})\cdot\mathbf{r}}\phi_i(\mathbf{r})\right|^2\delta\left(\frac{k^2}{2} - (\omega + \epsilon_i)\right), \tag{15}$$

where ω is the energy of the perturbation, $|\phi_{\mathbf{k}}\rangle$ the outgoing electron wavefunction, which in the second step we assume to be a planewave. In the long wavelength limit ($\mathbf{q} \to 0$) we can recognize the Fourier transform of the wavefunction $\tilde{\phi}_i(\mathbf{k}) = \int d\mathbf{r}e^{i(\mathbf{k})\cdot\mathbf{r}}\phi_i(\mathbf{r})$

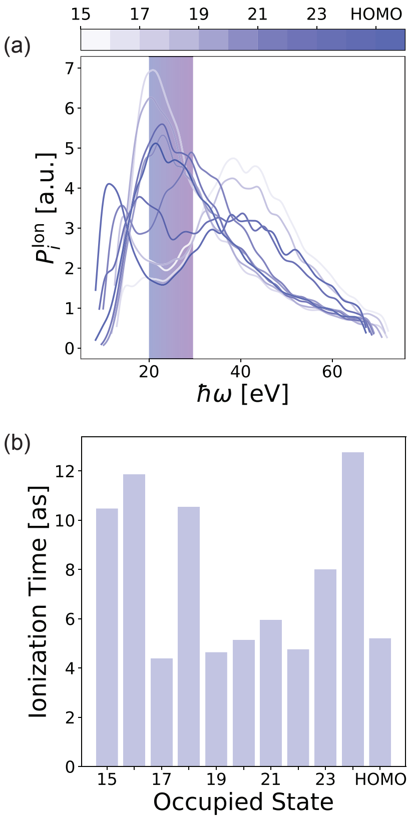

Figure S12. XUV-photoionisation rate for the occupied orbitals (a) calculated with Eq. (16). (b) Orbital dependent ionisation time calculated by integrating the curves in (a) within the XUV energy window.

and write:

$$P^{\mathrm{Ion}}_i(\omega) = \frac{2\pi}{V}\sum_{\mathbf{k}}\left|\tilde{\phi}_{i\mathbf{k}}\right|^2\delta\left(\frac{k^2}{2} - (\omega + \epsilon_i)\right). \tag{16}$$

Using this formula, we evaluated the orbital dependent ionisation probability for the 15 outer valence KS orbitals reported in Fig. S12(a).

By convoluting these photoionisation probabilities with an energy distribution function $f^{\mathrm{XUV}}(\omega)$ for the XUV pulse, we can get the orbital resolved ionisation rate as:

$$R^{\mathrm{XUV}}_c = \int d\omega\, f^{\mathrm{XUV}}(\omega)\,P^{\mathrm{Ion}}_c(\omega). \tag{17}$$

In our calculations we make the easiest assumption by choosing $f^{\mathrm{XUV}}(\omega)$ as a stepwise window function between 19.5 and 30.5 eV (as in the experiment) and extract the time it takes for a given valence orbital to be fully ionised as the inverse of the ionisation rates, see Fig. S12(b).

c. The shake-up process and the characteristic time delay

We can finally combine the XUV photoionisation probability with the state resolved shake-up rates and get the characteristic shake-up times. More specifically, the probability that an empty bound state gets populated by shake-up processes following XUV ionisation can be written as:

$$P_i^{\text{Sh-up}}(t) = 1 - e^{R_i^{\text{tot}} t} \tag{18}$$

where the total rate is given by:

$$R_i^{\text{tot}} = \sum_c \left(1 - e^{-R_c^{\text{XUV}} T^{\text{XUV}}}\right) R_{ic}^{\text{Sh-up}} \tag{19}$$

i.e. the probability of having a hole in state c times the rate of shake-up towards a state i summed over all initial holes. The probability of creating initial holes takes into account the actual duration of the XUV pulse T^{XUV}.

In Fig. S13(a) we show the characteristic electronic shake-up times as reported in the main text. For more thorough validation of this result we computed the same quantities as a function of the artificial parameter η. These results are reported in Fig. S13(b) for the final empty states ionised with two IR photons and it is apparent that the variation of the characteristic times with respect to η is small for any $\eta \geq 0.1$ eV. In our calculations we chose $\eta = 0.1$, which is safe to assume considering typical quasiparticle lifetimes in molecules[41]. Furthermore we choose this particular value as the smallest broadening such that the results can still be considered independent of the choice. In this way we avoid the inclusion of unrealistic shake-up processes due to a too loose energy matching criterion.

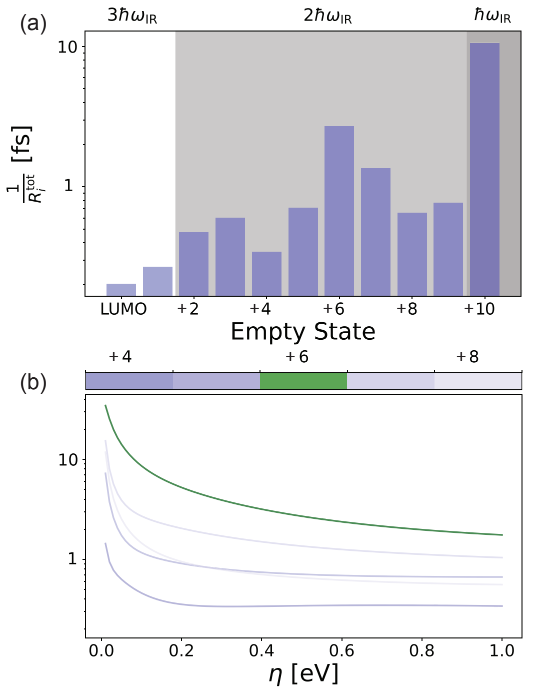

Figure S13. **Shake-up rates for the first unoccupied orbitals:** (a) Shake-up rates where the Dirac delta has been approximated by a Lorentzian with width $\eta = 0.1$ eV and a photoionisation time ($T_{\text{XUV}} = 100$ as). (b) Same rates as a function of the broadening parameter η.

10. Non-equilibrium Green's function approach

a. Theoretical description

In this section we discuss the details of the ab-initio real-time propagation presented in the main paper. We use the non-equilibrium Green's function (NEGF) formalism and solve numerically the Kadanoff–Baym equations (KBE's)[42–44] within the Generalized Kadanoff–Baym Ansatz (GKBA)[45].

Within the GKBA, the KBE's collapse into a single equation for the single-particle density matrix $\rho(t)$

$$\frac{d}{dt}\rho(t) + i[h_{\text{HF}}(t), \rho(t)] = -[I_{\text{corr}}(t) - I_{\text{ion}}(t) + \text{h.c.}]. \tag{20}$$

The left-hand side contains the commutator between ρ and the time-dependent Hartree-Fock (HF) Hamiltonian

$$h_{\text{HF}}(t) = h_{\text{KS}} - V_{\text{Hxc}} + V_{\text{HF}}(t) + \boldsymbol{\mathcal{E}}(t) \cdot \mathbf{d}\,, \tag{21}$$

where h_{KS} is the Kohn-Sham (KS) Hamiltonian, V_{Hxc} is the Hartree-exchange-correlation potential (both h_{KS} and V_{Hxc} are evaluated at the equilibrium KS density), $V_{\text{HF}}(t) = V_{\text{HF}}[\rho(t)]$ is the time-dependent HF potential, $\boldsymbol{\mathcal{E}}(t)$ is the external pulse, and $\mathbf{d} = (d_x, d_y, d_z)$ is the molecular dipole vector. Correlation effects are included in the right-hand side of Eq. (20)

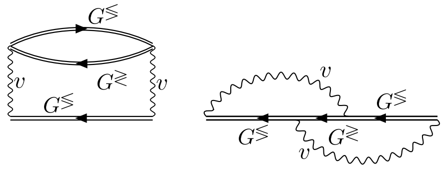

Figure S14. **Diagrammatic representation of the second Born self-energy**. The double line represents the Green's function $G^{\lessgtr}$ while the wiggly line represents the Coulomb interaction v.

via the *collision integral*

$$I_{\rm corr}(t) = \int_0^t dt' [\Sigma^>_{\rm corr}(t,t')G^<(t',t) - \Sigma^<_{\rm corr}(t,t')G^>(t',t)] \,, \qquad (22)$$

where $G^{\lessgtr}$ is the lesser (greater) non-equilibrium Green's function, and $\Sigma^{\lessgtr}_{\rm corr}$ is the correlation lesser (greater) non-equilibrium self-energy. Here we approximate $\Sigma_{\rm corr}$ at the second Born (2B) level, see Fig. S14 for its diagrammatic representation. The GKBA consists in approximating the lesser and greater Green's functions as

$$G^<(t,t') = -G^R(t,t')\rho(t') + \rho(t)G^A(t,t'), \qquad (23)$$

$$G^>(t,t') = G^R(t,t')\bar{\rho}(t') - \bar{\rho}(t)G^A(t,t'), \qquad (24)$$

where $\bar{\rho}(t) = 1 - \rho(t)$ and

$$G^R(t,t') = [G^A(t',t)]^\dagger = -i\theta(t-t')T[e^{-i\int_{t'}^t d\bar{t}\, h_{\rm HF}(\bar{t})}]. \qquad (25)$$

The second term appearing on the right-hand side of Eq. (20) is the *ionisation integral*

$$I_{\rm ion}(t) = \int_0^t dt' \Sigma^>_{\rm ion}(t,t')G^<(t',t) \,, \qquad (26)$$

with $\Sigma^>_{\rm ion}$ the ionisation self-energy. This term describes the excitation of bound electrons to continuum states occurring during the ionisation process induced by the pulse $\boldsymbol{\mathcal{E}}(t)$.

The numerical solution of Eq. (20) with $\Sigma_{\rm corr}$ in the 2B approximation scales quadratically with the maximum propagation time. This favourable scaling (in comparison to the cubic scaling of the KBE) allows us to follow the dynamics of molecules with $\sim 10^2$ active electrons for tens of femtoseconds.

b. Computational method

In this section we discuss how Eq. (20) is practically solved to study the ultrafast electron dynamics of adenine after the action of an XUV pulse. We first obtain the KS ground state (GS) of the molecule using the Octopus code[27], with HSCV pseudopotentials[46], the PBE approximation for the exchange-correlation potential[47] and the averaged density self-interaction correction (ADSIC)[39]. The grid is a sphere of radius 20 Bohr with spacing 0.25 Bohr. The lowest 57 KS orbitals are bound and constitute the *active space* to describe the molecular ρ. The remaining KS orbitals (with KS energy $\epsilon_k^{\rm KS} > 0$) up to energies ~ 41 eV (about 3000 states) are instead used to build $\Sigma_{\rm ion}$.

We then solve the equilibrium HF problem (with $\boldsymbol{\mathcal{E}} = 0$) in the active space. Since correlation effects are typically weak in the ground state of biomolecules[48–50], we set $\rho(0) = \rho_{\rm HF}$ as initial condition for Eq. (20). It is therefore convenient to work in the HF basis $\{\varphi_i^{\rm HF}(\mathbf{r})\}$. In this basis the equilibrium HF Hamiltonian $h_{\rm HF}$ is diagonal and the 2B self-energy reads

$$\Sigma^{\lessgtr}_{{\rm corr},ij}(t,t') = \sum_{nmpqsr} v_{irpn}(2v_{mqsj} - v_{mqjs}) \times G^{\lessgtr}_{nm}(t,t')G^{\lessgtr}_{pq}(t,t')G^{\gtrless}_{sr}(t',t), \qquad (27)$$

where the Coulomb integrals v_{irpn} are computed like in Eq. (10) with the HF basis functions. The correlation self-energy in Eq. (27) is numerically evaluated using the *dissection algorithm*[51] implemented in the CHEERS code[32]. Additionally only those Coulomb integrals with at most one index above the HF-HOMO are retained, see Fig. S15. This amounts to including only shake-up processes for electrons visiting HF states initially unoccupied. A similar approximation has been recently used in the context of Auger decays[33], finding excellent agreement with Configuration Interaction calculations. The approximation reduces the number of Coulomb integrals involved in $\Sigma_{\rm corr}$ by about an order of magnitude: from $N_{\rm tot}^4$, where $N_{\rm tot} = 57$ is the dimension of the active space, to $N_{\rm occ}^4 + 4N_{\rm occ}^3(N_{\rm tot} - N_{\rm occ})$, where $N_{\rm occ} = 27$ is the number of initially occupied states. Furthermore, $\rho(0) = \rho_{\rm HF}$ is a stationary solution of Eq. (20) in the absence of external fields.

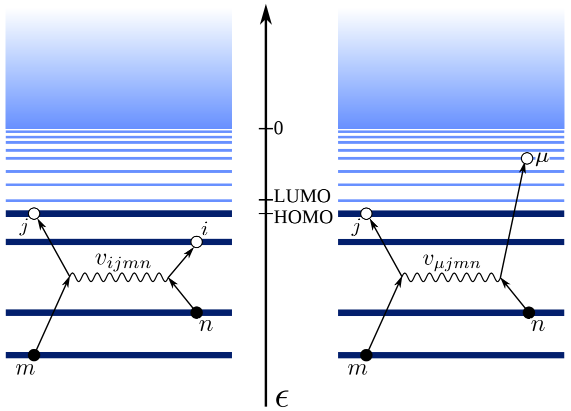

Figure S15. **Schematic illustration of scattering processes**. Left: scattering involving electrons in initially occupied states (labelled by roman indices). Right: shake-up scatterings leading to the transition of an electron in an initially unoccupied state (labeled by μ). The initially occupied and unoccupied states are represented by a dark blue and a light blue line, respectively. The shaded area represents the continuum – no scattering is considered in this area.

The ionisation self-energy in the HF basis reads

$$\Sigma^{>}_{\text{ion},ij}(t,t') = -i \sum_{k:\ \varepsilon_k^{\text{KS}}>0} [\boldsymbol{\mathcal{E}}(t)\cdot \mathbf{d}_{ik}] e^{-i\epsilon_k^{\text{KS}}(t-t')} [\boldsymbol{\mathcal{E}}(t')\cdot \mathbf{d}_{kj}], \tag{28}$$

where

$$\mathbf{d}_{ij} = \int d\mathbf{r}\ \varphi_i^{\text{HF}}(\mathbf{r})\mathbf{r}\varphi_j^{\text{HF}}(\mathbf{r}) \tag{29}$$

and $\varepsilon_k^{\text{KS}} = \varepsilon_k^{\text{HF}}$ since we discard the Coulomb repulsion of HF electrons in the continuum. The main physical process left out by this further approximation is the Auger decay which, however, can be safely ignored for XUV pulses. We also emphasise that the ionisation self-energy in Eq. (28) is suitable only for single-photon ionisation processes (like the ones induced by the XUV pulse of the present experiment). The inclusion of multiphoton processes would lead to a different dependence of Σ_{ion} on $\boldsymbol{\mathcal{E}}$.

The NEGF-GKBA Eq. (20) is solved numerically using the CHEERS code[32]. From the single-particle density matrix we can easily calculate the time-dependent electronic density according to

$$n_{\text{el}}(\mathbf{r},t) = \sum_{ij} \varphi_i^{\text{HF}}(\mathbf{r})\rho_{ij}(t)\varphi_i^{\text{HF}}(\mathbf{r}). \tag{30}$$

c. Ionisation

In our simulations we have studied the electron dynamics of adenine under the influence of the experimental XUV laser pulse. The latter has a full-width at half-maximum of ~ 300 as and a central frequency of ~ 27 eV (see the inset of Fig. S16 for the temporal shape of the pulse and for its power spectrum). In Fig. S16 we show the time-dependent variation of the occupations $\delta n_i(t) = n_i(t) - n_i(0)$ of the bound KS orbitals $\{\varphi_i^{\text{KS}}\}$ during the ionisation process. Exciting the molecule with a realistic pulse allows us to take into account the laser induced electronic coherence. In refs.[52,53], where an equivalent theoretical approach is used, the effect of laser induced coherence is shown to affect the evolution of charge migration. The occupations $n_i(t)$ are obtained according to

$$n_i(t) = \sum_{mn} U_{im}\rho_{mn}(t)U^{\dagger}_{ni}, \tag{31}$$

where $U_{im} = \int d\mathbf{r}\ \varphi_i^{\text{KS}}(\mathbf{r})\varphi_m^{\text{HF}}(\mathbf{r})$ is the change of basis transformation matrix.

The inspection of Fig. S16 shows that all initially occupied (unoccupied) KS states depopulate (populate) with superimposed oscillations following the cycles of the laser pulse. We point out that the initially unoccupied KS states get populated mainly through ultrafast shake-up processes. Longer timescale dynamics are discussed in the main paper.

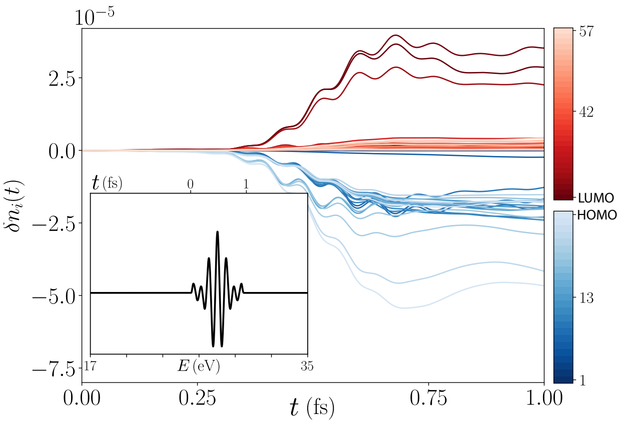

Figure S16. **Time-dependent variation of the population**. Change of the population of the occupied (blue) and unoccupied (red) bound KS states of adenine excited by the XUV pulse perpendicular to the plane of the molecule. The inset shows the spectrum and the temporal profile of the pump pulse.

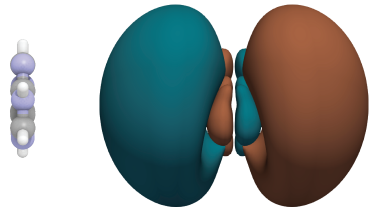

Figure S17. **Shape of the LUMO+6 orbital.** The lobes extend perpendicularly from the molecular plane.

d. Charge migration

As stated in the main text, the simulations reveal an inflation of the electron density of the molecule over time, caused by the population of the particular KS state LUMO+6 (Figure S17) on a timescale compatible with the delay observed in the yield of stable adenine dication. The inflation indicates that the electrons move away from the molecule over time. Defining a slab around the molecular plane of thickness $d = 6$ Å and integrating the electron density outside of this area, we have a measure of the molecular inflation. In Fig. 3(b) of the main text, we show how this integrated electron density reflects the timescale of the inflation, as expected. The observed behaviour is consistent for different slab thicknesses. This effect is due to the population of the delocalized bound states, as reported in the main text, and in fact has the two characteristic time scales observed in the population of the initially unoccupied KS states, the slower one being the one associated with the delayed shake-up transition.

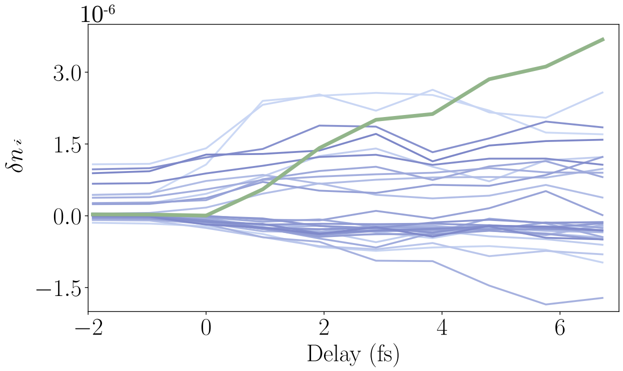

Figure S18. **Shake-up state depletion.** Average shake-up states depletion as a function of XUV-NIR delay. The average has been performed in a window of 1 fs after the end of the pulses. In accordance to the main text, the LUMO+6 is drawn in green while the rest in shades of light blue.

11. NIR-induced ionisation

To support the analysis on the role of the LUMO+6 state in the delayed appearance of the doubly ionized adenine molecule in the experimental measurement, we calculated the dynamics of the system when the additional NIR pulse is introduced. In our simulation we have used a pulse with carrier frequency 1.7 eV and $\sin^2$ envelope of total duration 200a.u. $\simeq$ 4.8 fs. Of particular interest is the time-dependent occupation of the shake-up states as the NIR pulse acts on the system. In fig. S18 the total NIR-induced state depletion for the different shake-up states as a function of XUV-NIR delay is shown. These are defined as the averaged value of the NIR-induced variation of the state occupation in a window of 1 fs after the end of the NIR pulse. As stated in the main paper the NIR-induced depletion of the LUMO+6 state has an onset of 2–4 fs and reproduces the behaviours of the adenine dication yield. Moreover, the LUMO+6 state is the only one, of all the shake-up states, showing these characteristics.

Supplementary References

[1] Drescher, M. *et al.* Time-resolved atomic inner-shell spectroscopy. *Nature* **419**, 803–807 (2002).

[2] Hanna, A. M., Vendrell, O., Ourmazd, A. & Santra, R. Laser control over the ultrafast Coulomb explosion of N_2^{2+} after Auger decay: A quantum-dynamics investigation. *Physical Review A* **95**, 043419 (2017).

[3] Averbukh, V. *et al.* Interatomic electronic decay processes in singly and multiply ionized clusters. *Journal of Electron Spectroscopy and Related Phenomena* **183**, 36–47 (2011). Electron Spectroscopy Kai Siegbahn Memorial Volume.

[4] Cederbaum, L. S. & Zobeley, J. Ultrafast charge migration by electron correlation. *Chemical Physics Letters* **307**, 205–210 (1999).

[5] Kuleff, A. I., Lünnemann, S. & Cederbaum, L. S. Electron-correlation-driven charge migration in oligopeptides. *Chemical Physics* **414**, 100–105 (2013). Attosecond spectroscopy.

[6] Kuleff, A. I. & Cederbaum, L. S. Ultrafast correlation-driven electron dynamics. *Journal of Physics B: Atomic, Molecular and Optical Physics* **47**, 124002 (2014).

[7] Remacle, F. & Levine, R. D. An electronic time scale in chemistry. *Proceedings of the National Academy of Sciences* **103**, 6793–6798 (2006). https://www.pnas.org/content/103/18/6793.full.pdf.

[8] Calegari, F., Sansone, G., Stagira, S., Vozzi, C. & Nisoli, M. Advances in attosecond science. *Journal of Physics B: Atomic, Molecular and Optical Physics* **49**, 062001 (2016).

[9] Nisoli, M., Decleva, P., Calegari, F., Palacios, A. & Martín, F. Attosecond electron dynamics in molecules. *Chemical Reviews* **117**, 10760–10825 (2017).

[10] Calegari, F. *et al.* Ultrafast electron dynamics in phenylalanine initiated by attosecond pulses. *Science* **346**, 336–339 (2014).

[11] Lara-Astiaso, M. *et al.* Attosecond pump–probe spectroscopy of charge dynamics in tryptophan. *The Journal of Physical Chemistry Letters* **9**, 4570–4577 (2018).

[12] Stolow, A. The three pillars of photo-initiated quantum molecular dynamics. *Faraday Discuss.* **163**, 9–32 (2013).

[13] Jenkins, A. J., Vacher, M., Bearpark, M. J. & Robb, M. A. Nuclear spatial delocalization silences electron density oscillations in 2-phenyl-ethyl-amine (pea) and 2-phenylethyl-n,n-dimethylamine (penna) cations. *The Journal of Chemical Physics* **144**, 104110 (2016).

[14] Polyak, I. *et al.* Charge migration engineered by localisation: electron-nuclear dynamics in polyenes and glycine. *Molecular Physics* **116**, 2474–2489 (2018).

[15] Lara-Astiaso, M. *et al.* Decoherence, control and attosecond probing of XUV-induced charge migration in biomolecules. a theoretical outlook. *Faraday Discuss.* **194**, 41–59 (2016).

[16] Marciniak, A. *et al.* Electron correlation driven non-adiabatic relaxation in molecules excited by an ultrashort extreme ultraviolet pulse. *Nature Communications* **10**, 337 (2019).

[17] Hervé, M. *et al.* Ultrafast dynamics of correlation bands following XUV molecular photoionization. *Nature Physics* (in press, 16 November) (2020).

[18] Corkum, P. B. Plasma perspective on strong field multiphoton ionization. *Physical Review Letters* **71**, 1994–1997 (1993).

[19] Sola, I. J. *et al.* Controlling attosecond electron dynamics by phase-stabilized polarization gating. *Nature Physics* **2**, 319–322 (2006).

[20] Pilling, S. *et al.* Dissociative photoionization of adenine following valence excitation. *Rapid Communications in Mass Spectrometry* **21**, 3646–3652 (2007).

[21] Alvarado, F., Bari, S., Hoekstra, R. & Schlathölter, T. Interactions of neutral and singly charged keV atomic particles with gas-phase adenine molecules. *The Journal of Chemical Physics* **127**, 034301 (2007).

[22] Brédy, R. *et al.* Fragmentation of adenine under energy control. *The Journal of Chemical Physics* **130**, 114305 (2009).

[23] van der Burgt, P. J. M., Finnegan, S. & Eden, S. Electron impact fragmentation of adenine: partial ionization cross sections for positive fragments. *The European Physical Journal D* **69**, 173 (2015).

[24] Galbraith, M. C. E. *et al.* Few-femtosecond passage of conical intersections in the benzene cation. *Nature Communications* **8**, 1018 (2017).

[25] Marques, M. A., Castro, A., Bertsch, G. F. & Rubio, A. octopus: a first-principles tool for excited electron–ion dynamics. *Computer Physics Communications* **151**, 60–78 (2003).

[26] Castro, A. *et al.* octopus: a tool for the application of time-dependent density functional theory. *Physica Status Solidi B* **243**, 2465–2488 (2006).

[27] Andrade, X. *et al.* Real-space grids and the octopus code as tools for the development of new simulation approaches for electronic systems. *Physical Chemistry Chemical Physics* **17**, 31371–31396 (2015).

[28] Runge, E. & Gross, E. K. U. Density-functional theory for time-dependent systems. *Physical Review Letters* **52**, 997–1000 (1984).

[29] Bertsch, G. F., Iwata, J. I., Rubio, A. & Yabana, K. Real-space, real-time method for the dielectric function. *Physical Review B* **62**, 7998–8002 (2000).

[30] Alonso, J. L. *et al.* Efficient formalism for large-scale *ab initio* molecular dynamics based on time-dependent density functional theory. *Physical Review Letters* **101**, 096403 (2008).

[31] Cucinotta, C. S., Hughes, D. & Ballone, P. Real-time real-space TD-DFT for atoms: Benchmark computations on a nonspherical logarithmic grid. *Physical Review B* **86**, 045114 (2012).

[32] Perfetto, E. & Stefanucci, G. CHEERS: a tool for correlated hole-electron evolution from real-time simulations. *Journal of Physics: Condensed Matter* **30**, 465901 (2018).

[33] Covito, F., Perfetto, E., Rubio, A. & Stefanucci, G. Real-time dynamics of Auger wave packets and decays in ultrafast charge migration processes. *Physical Review A* **97**, 061401 (2018).

[34] Månsson, E. P. *et al.* Ultrafast dynamics in the DNA building blocks thymidine and thymine initiated by ionizing radiation. *Phys. Chem. Chem. Phys.* **19**, 19815–19821 (2017).

[35] Seiffert, L. *et al.* Attosecond chronoscopy of electron scattering in dielectric nanoparticles. *Nature Physics* **13**, 766–770 (2017).

[36] Plützer, C., Nir, E., de Vries, M. S. & Kleinermanns, K. IR–UV double-resonance spectroscopy of the nucleobase adenine. *Physical Chemistry Chemical Physics* **3**, 5466–5469 (2001).

[37] Eppink, A. T. J. B. & Parker, D. H. Velocity map imaging of ions and electrons using electrostatic lenses: Application in photoelectron and photofragment ion imaging of molecular oxygen. *Review of Scientific Instruments* **68**, 3477–3484 (1997).

[38] Lacoursière, J., Meyer, M., Nahon, L., Morin, P. & Larzillière, M. Time-resolved pump-probe photoelectron spectroscopy of helium using a mode-locked laser synchronized with synchrotron radiation pulses. *Nuclear Instruments and Methods in Physics Research Section A: Accelerators, Spectrometers, Detectors and Associated Equipment* **351**, 545–553 (1994).

[39] Legrand, C., Suraud, E. & Reinhard, P.-G. Comparison of self-interaction-corrections for metal clusters. *Journal of Physics B: Atomic, Molecular and Optical Physics* **35**, 1115 (2002).

[40] Andrade, X. *et al.* Modified Ehrenfest formalism for efficient large-scale ab initio molecular dynamics. *Journal of Chemical Theory and Computation* **5**, 728–742 (2009).

[41] Caruso, F., Rinke, P., Ren, X., Rubio, A. & Scheffler, M. Self-consistent *gw*: All-electron implementation with localized basis functions. *Physical Review B* **88**, 075105 (2013).

[42] Kadanoff, L. P. & Baym, G. A. *Quantum statistical mechanics: Green's function methods in equilibrium and nonequilibrium problems* (Benjamin, 1962).

[43] Stefanucci, G. & van Leeuwen, R. *Nonequilibrium Many-Body Theory of Quantum Systems: A Modern Introduction* (Cambridge University Press, Cambridge, 2013).

[44] Balzer, K. & Bonitz, M. *Nonequilibrium Green's Functions Approach to Inhomogeneous Systems* (Springer, 2012).

[45] Lipavský, P., Špička, V. & Velický, B. Generalized kadanoff-baym ansatz for deriving quantum transport equations. *Physical Review B* **34**, 6933–6942 (1986).

[46] Vanderbilt, D. Optimally smooth norm-conserving pseudopotentials. *Physical Review B* **32**, 8412–8415 (1985).

[47] Perdew, J. P., Burke, K. & Ernzerhof, M. Generalized gradient approximation made simple. *Physical Review Letters* **77**, 3865–3868 (1996).

[48] Kuleff, A. I., Breidbach, J. & Cederbaum, L. S. Multielectron wave-packet propagation: General theory and application. *The Journal of Chemical Physics* **123**, 044111 (2005).

[49] Kuleff, A. I. & Cederbaum, L. S. Charge migration in different conformers of glycine: The role of nuclear geometry. *Chemical Physics* **338**, 320–328 (2007). Molecular Wave Packet Dynamics.

[50] Ruberti, M., Decleva, P. & Averbukh, V. Multi-channel dynamics in high harmonic generation of aligned CO2: ab initio analysis with time-dependent b-spline algebraic diagrammatic construction. *Phys. Chem. Chem. Phys.* **20**, 8311–8325 (2018).

[51] Perfetto, E. & Stefanucci, G. The dissection algorithm for the second-born self-energy. *Physica Status Solidi B* **256**, 1800573 (2019).

[52] Perfetto, E., Sangalli, D., Marini, A. & Stefanucci, G. Ultrafast charge migration in xuv photoexcited phenylalanine: A first-principles study based on real-time nonequilibrium Green's functions. *The Journal of Physical Chemistry Letters* **9**, 1353–1358 (2018).

[53] Perfetto, E. *et al.* Ultrafast quantum interference in the charge migration of tryptophan. *The Journal of Physical Chemistry Letters* **11**, 891–899 (2020).
